# Medical examiners, coroners, and bioterrorism.

**DOI:** 10.3201/eid0605.000522

**Published:** 2000

**Authors:** K B Nolte, S S Yoon, C Pertowski


### Medical Examiners, Coroners, and Bioterrorism

**To the Editor:** Federal, state, and local agencies are developing plans to detect and respond to bioterrorism. Medical examiners and coroners should be included in these plans. A multifaceted response team for bioterrorist events includes health-care providers and law enforcement, public health, and public safety officials. Since medical examiners and coroners generally work independently from other members of this team, special efforts may be necessary to ensure their inclusion in the planning process.

Medical examiners and coroners have state statutory authority to investigate violent, suspicious, sudden, or unexplained deaths (1), including those due to homicide, trauma, and inapparent or poorly explained causes, such as drugs, toxins, and infectious agents. The role of these medical professionals in bioterrorism response can be twofold: response to a known terrorist attack and surveillance for unusual deaths or clusters of deaths that may represent an undetected attack. Deaths from terrorism are homicides and therefore under the jurisdiction of medical examiners and coroners. These investigators are skilled in preserving medicolegal evidence that may be important for subsequent criminal proceedings and in handling situations that involve mass deaths, as shown by their participation in the investigations of the Oklahoma City bombing, aviation accidents, and heat-related deaths (2-4).

Medical examiners and coroners may also play an important role in the detection of bioterrorism since they may recognize unusual deaths before health-care providers become involved. Patients who die of infectious diseases or poisoning often die at home (5,6). Even patients who come to a health-care facility for treatment may die precipitously and unexpectedly, without a clear diagnosis, and may come under the jurisdiction of medical examiners and coroners. For example, in the 1993 outbreak of hantavirus pulmonary syndrome in the southwestern United States, medical examiners played an important role in recognizing the novel, rapidly fatal infectious syndrome (7). Autopsies are an effective way of obtaining an accurate diagnosis for deaths from infectious diseases and toxic exposures. In 1979, autopsy pathologists played a critical role in recognizing inhalational anthrax cases caused by an accidental discharge from a former Soviet bioweapons laboratory (8). In 1985, Illinois medical examiners identified the cause of death of persons who ingested acetaminophen that had been intentionally contaminated with cyanide (9).

To be fully integrated into the medical, law enforcement, and public health plans for detecting and responding to bioterrorism, medical examiners and coroners will need information about the biological and chemical agents likely to be used, access to laboratories capable of identifying these agents, adequate data management systems for mortality surveillance, and improved autopsy facilities and procedures to ensure that prosectors are protected from infectious and chemical agents. As a beginning, the Centers for Disease Control and Prevention recently funded a model medical examiner surveillance program for bioterrorism mortality in New Mexico. Further collaboration among federal, state, and local agencies and medicolegal death investigators will be required for the components of such a program to be effective on a national scale.

**Kurt B. Nolte,*† Stephen S. Yoon,† and Carol Pertowski†**
*University of New Mexico School of Medicine, Albuquerque, New Mexico, USA; †Centers for Disease Control and Prevention, Atlanta, Georgia, USA

### References

1. Combs DL, Parrish RG, Ing R. Death investigation in the United States and Canada, 1995. Atlanta (GA): Centers for Disease Control and Prevention; 1995.
2. Jordan FB. The role of the medical examiner in mass casualty situations with special reference to the Alfred P. Murrah Building bombing. J Okla State Med Assoc 1999;92:159-63.
3. McCarty VO, Sohn AP, Ritzlin RS, Gauthier JH. Scene investigation, identification, and victim examination following the accident of Galaxy 203: disaster preplanning does work. J Forensic Sci 1987;32:983-7.
4. Centers for Disease Control and Prevention. Heat-related mortality—Chicago, July 1995. MMWR Morb Mortal Wkly Rep 1995;44:577-9.
5. Soslow AR, Woolf AD. Reliability of data sources for poisoning deaths in Massachusetts. Am J Emerg Med 1992;10:124-7.
6. Luke JL, Halpern M. Sudden unexpected death from natural causes in young adults. Arch Pathol 1968;85:10-77.

7. Nolte KB, Simpson GL, Parrish RG. Emerging infectious agents and the forensic pathologist: the New Mexico model. Arch Pathol Lab Med 1996;120:125-8.
8. Walker DH, Yampolska O, Grinberg LM. Death at Sverdlovsk: what have we learned? Am J Pathol 1994;144:1135-41.
9. Lifschultz BD, Donoghue ER. The Tylenol cyanide poisonings. American Academy of Forensic Sciences 1989;46(Abstract A12).

### Seroprevalence of Human Hantavirus Infection in the Ribeirão Preto Region of São Paulo State, Brazil

**To the Editor:** Hantavirus pulmonary syndrome (HPS) has been identified in the region of Ribeirão Preto, São Paulo State, Brazil, since 1993 (1-4). As of September 1999, 38 HPS cases had been reported in Brazil, 16 in the state of São Paulo (2). Between May 1998 and August 1999, the Adolfo Lutz Institute (ALI) in São Paulo city serologically confirmed five cases—three fatal—in the Ribeirão Preto region: two from Guariba, one from Jardinópolis, one from Cajuru, and one from Cassia dos Coqueiros (Luiza Teresinha Madia de Souza, ALI, pers. comm.).

Despite these reports and suspicions of additional cases, the prevalence of hantavirus infection and HPS in the region is not known. Laboratory confirmation has not been available locally, and sending serum samples to ALI for laboratory evaluation is not feasible in most cases. Thus, only presumptive diagnoses could be made until the Sin Nombre virus (SNV) enzyme-linked immunosorbent assay (ELISA) was developed.

To estimate the occurrence and distribution of human hantavirus infection, we used SNV ELISA to conduct a serologic survey of a sample of hospital patients requiring venipuncture for routine procedures. The patients came from three regional cities: Ribeirão Preto, Guariba, and Jardinópolis. Between February and June 1999, a total of 567 samples were collected: 257 from the public hospital of Guariba, 110 from the public hospital in Jardinópolis, and 200 from the General Hospital of the School of Medicine of Ribeirão Preto. When we compared our sample with the general population, the patients in the study sample were slightly older but similar in sex distribution.

Sixteen additional samples were evaluated to confirm the effectiveness of SNV ELISA in diagnosing hantavirus infection: 12 from patients in whom HPS was clinically suspected and 4 previously confirmed by ALI in the city of São Paulo between May 1998 and August 1999. Known HPS convalescent-phase plasma provided by the Centers for Disease Control and Prevention (CDC) was used as positive control. Negative controls were selected by simple random sampling from all previously negative samples.

Positive and negative recombinant SNV antigens provided by CDC were coated on microtiter plates at 1:2,000 dilution in phosphate-buffered saline overnight at 4°C and used in a standard immunoglobulin G testing format. Reverse transcriptase-polymerase chain reaction analysis of serum from two fatal cases of HPS occurring in the cities of Franca and Araraquara suggested the presence of two genetically distinct hantaviruses in the area surrounding Ribeirão Preto. Antigen prepared from local virus is not considered to be necessary for immunoassays because the local virus is not sufficiently different from other isolates to require special antigen preparation (5).

All samples were screened in duplicate on both positive and negative antigens in the assay. A sample was considered positive if absorbance on the positive antigen was greater than absorbance on both the negative control antigen and the negative control of the plate. To confirm the diagnosis, samples satisfying these criteria were tested in duplicate along with 14 negative samples. Samples were considered positive when their subtractive absorbance was greater than the calculated mean subtractive absorbance of the 14 negative samples and three standard deviations.

From our serologic survey, the seroprevalence of human hantavirus infection was determined to be 1.23% (7/567) overall, 0.5% (1/200) in Ribeirão Preto, 0.4% (1/257) in Guariba, and 4.5% (5/110) in Jardinópolis. If one assumes the inhabitants sampled were representative, the seroprevalence provides an estimate of surviving past or recent hantavirus infections in the area. As the overall antibody prevalence of 1.23% is more than twice that observed in the U.S. populations at risk for hantavirus infection, such infections are not rare in the Ribeirão Preto region (6).

